# Prognostic relevance of Bmi-1 expression and autoantibodies in esophageal squamous cell carcinoma

**DOI:** 10.1186/1471-2407-10-467

**Published:** 2010-09-01

**Authors:** Wan-li Liu, Xian-zhi Guo, Lan-jun Zhang, Jun-ye Wang, Ge Zhang, Su Guan, Yu-min Chen, Qing-li Kong, Li-hua Xu, Man-zhi Li, Li-bing Song, Mu-sheng Zeng

**Affiliations:** 1 Department of Experimental Research, Sun Yat-sen University cancer center, Guangzhou, China; 2 State Key Laboratory of Oncology in South China, Guangzhou, China; 3 Department of Medical Oncology, The Central Hospital of Xuhui District, Shanghai, China; 4 Department of Thoracic Surgery, Sun Yat-sen University cancer center, Guangzhou, China; 5 School of Pharmaceutical Sciences, Sun Yat-sen University, Guangzhou, China

## Abstract

**Background:**

Overexpression of Bmi-1 has been observed in a variety of cancers, and it has been suggested to be an independent prognostic marker for the patients. The objective of this study was to determine the level of Bmi-1 expression or its autoantibodies in human esophageal squamous cell carcinoma (ESCC) and to correlate it with clinicopathologic data.

**Methods:**

We first examined Bmi-1 expression in ESCC cell lines and tumor samples by RT-PCR and Western blot analysis. We then analyzed Bmi-1 protein expression in 171 clinicopathologically characterized ESCC cases by immunohistochemistry. In addition, we detected its autoantibodies in sera of patients with ESCC by ELISA.

**Results:**

We found that Bmi-1 expression was higher in the immortalized cells, cancer cell lines and most cancer tissue than in non-tumorous control tissue at both mRNA and protein level. In addition, Bmi-1 expression was observed in 64.3% (110 of 171) archive ESCC specimen by immunohistochemistry analysis, and the location of Bmi-1 in ESCC was in the nuclei instead of cytoplasm of tumor cells. There was a significant difference of Bmi-1 expression in patients categorized according to stage (*P *= 0.003) and pN classification (*P *= 0.047). Multivariate analysis suggested that Bmi-1 expression was an independent prognostic marker for ESCC patients. A prognostic significance of Bmi-1 was also found in the subgroup of T3~T4 and N1 tumor classification. Bmi-1 autoantibodies were detected in sera of 39.0% (62 of 159) ESCC patients. The correlations between anti-Bmi-1 antibodies and tumor stage (*P *= 0.040), or lymph node status (*P *< 0.001) were significant.

**Conclusions:**

Our results suggest that Bmi-1 protein is a valuable marker of ESCC progression. The presence of Bmi-1 autoantibodies in sera from patients with ESCC may have clinical utility in esophageal cancer diagnosis.

## Background

Esophageal squamous cell carcinoma (ESCC), the major histological type of esophageal cancer, is the sixth most frequent cause of cancer death worldwide[1], and accounts for the fourth largest number of cancer death in China[2]. However, the molecular mechanism of its development and progression remains poorly understood[3]. Despite considerable diagnostic and therapeutic advances in the treatment of ESCC in recent years[4], there is still an urgent need for further identification of novel molecular markers to provide the clinician with useful information concerning patient prognosis and possible therapeutic options. Several factors, such as cyclin D1[5], Ki-67[6], nm23-H1[7], Fas[8] and CENP-H[9] have been reported previously as potentially useful prognostic markers in ESCC.

*Bmi-1 *(B-cell-specific Moloney murine leukemia virus integration site 1) was originally isolated as an oncogene that cooperates with *c-myc *in the generation of mouse pre B-cells lymphomas[10,11]. It is a transcriptional repressor belonging to the Polycomb-group (PcG) family of proteins involved in axial patterning, hematopoiesis, regulation of proliferation, and senescence[12,13]. It has been reported that Bmi-1 contributes to cell cycle regulation by acting as a stable transcriptional repressor of the INK4a/ARF locus[14]. Bmi-1 overexpression leads to activation of human telomerase reverse transcriptase transcription and induction of telomerase activity in immortalized mammary epithelial cells[15]. We have also reported that overexpression of Bmi-1 leads to the induction of telomerase activity, reduction of p16INK4a expression, and immortalization of normal nasopharygeal epithelial cells (NPECs)[16]. A recent report has shown that Bmi-1 autoantibodies were newly potential biomarkers of nasopharyngeal cancer[17]. In addition, it has been found that Bmi-1 is overexpressed in a variety of human cancers, such as mantle cell lymphomas[18], non-small cell lung cancer[19], B-cell non-Hodgkin's lymphoma[20], breast cancer[21], colorectal cancer[22], prostate cancer[23], nasopharyngeal carcinoma[16] and gastric carcinoma[24]. In these reports, Bmi-1 protein mainly locates in nuclei of tumor cells. Recently, He et al.[25] reported that Bmi-1 was overexpressed in esophageal squamous cell carcinomas, and Bmi-1 mRNA expression correlated with lymph node metastases, pathological stage and poor prognosis of the patients. However, they found that Bmi-1 protein was largely distributed in the cytoplasm of tumour cells, and there was no significant clinical relevance with Bmi-1 protein expression. Thus, it is required further investigation to determine whether the cytoplasm staining represents the real localization of Bmi-1, and whether Bmi-1 plays a different role in the development of ESCC.

Here, we found that overexpression of Bmi-1 was observed in both ESCC cell lines and tumor tissue. Moreover, the location of Bmi-1 in ESCC was in the nuclei instead of cytoplasm of tumor cells. The expression of Bmi-1 was correlated with the stage and pN classification of the disease. Multivariate analysis suggested that Bmi-1 expression was an independent prognostic marker for ESCC patients. In addition, Bmi-1 autoantibodies were presented in sera from patients with ESCC and weren't detected in sera from healthy control. Our results strongly suggest that the expression level of Bmi-1 might be used as a valuable prognostic marker for ESCC patients and Bmi-1 autoantibodes in serum may have potential clinical utility in ESCC diagnosis.

## Methods

### Cell lines

The primary esophageal epithelial cells was generated as described and cultured in Keratinocyte-SFM (Invitrogen, Carlsbad, CA) [3]. The immortalized esophageal epithelial cell line NE-3 induced by human papillomavirus type 16 E6/E7 and the ESCC cell line 108CA were obtained from Dr. Jin (the University of Hong Kong, P. R. China) and were cultured in Keratinocyte-SFM (Invitrogen, Carlsbad, CA) [3,9]. The ESCC cell lines Eca-109, TE-1, and Kyse140 (Cell Bank of Type Culture Collection of Chinese Academy of Sciences, Shanghai, China) were grown in RPMI 1640 (Invitrogen) supplemented with 10% fetal bovine serum[9].

### Sera

A group of 159 patients with ESCC who underwent tumor resection at the cancer center of Sun Yet-sen university from January 2005 to January 2007 was enrolled in this study. This group included 96 males and 63 females, with age ranging from 42 to 87 years (mean, 63 years). Sera of the patients were obtained at the time of diagnosis before treatment. Sera from 102 healthy volunteers, including 75 males and 27 females with ages from 41 to 71 years (mean, 58 years), were collected and used as control. Prior to the use of these sera for investigation, informed consent of patients and approval from the Institute Research Ethics Committee were obtained. After collection, sera were aliquoted and stored at -80°C until use.

### Tissue specimen

Eight pairs of ESCC tissue specimen and corresponding nontumorous specimen were obtained from patients with ESCC who underwent surgical esophageal tissue resection at the Cancer Center of Sun Yat-sen University (Guangzhou, P. R. China) during 2007. Written informed consent was obtained from each patient before surgery. All excised samples were obtained within 1 h after the operation from tumor tissue and corresponding nontumorous tissue 5-10 cm away from the tumor, and then were immediately kept in liquid nitrogen until further analysis. In addition, immunohistochemstry analysis was conducted on 171 paraffin-embedded ESCC samples which were histologically and clinically diagnosed from the Cancer Center, Sun Yat-sen University, between 2001 and 2004. None of them had received radiation therapy or chemotherapy before surgery. Prior to the use of these clinical materials for investigation, informed consent from patients and approval from the Institute Research Ethics Committee were obtained). Primary cancers of the esophagus were classified according to the pathological TNM classification[26]. Clinical information of 171 ESCC samples was described in detail as shown in Table 1. Patients included 129 males and 42 females, of ages ranging from 33 to 82 years (mean, 56.7 years). The figures on metastasis pertain to its presence at any time in follow-up. The median follow-up period for overall survival was 25.0 months for patients still alive at the time of analysis, and it was ranged from 1 to 78 months. A total of 112 (65.5%) patients died during follow-up period. Prior to the use of all of the clinical materials for investigation, informed consent from patients and approval from the Institute Research Ethics Committee were obtained.

**Table 1:** Correlation between the clinicopathologic features and expression of Bmi-1 protein

Characteristics	Bmi-1 expression		P
		
	No or low	High	
Age(y)	55.8 ± 10.4	57.6 ± 9.9	0.576
Gender			
Male	61(47.3)	68(52.7)	0.970
Female	20(47.6)	22(52.4)	
Stage			
I-IIa	50(58.8)	35(41.2)	0.003
IIb-IV	31 (36.0)	55(64.0)	
Histological differentiation			
Well	31(56.4)	24(43.6)	
Moderate	29(40.3)	43(59.7)	0.315
Poor	21(47.7)	23(52.3)	
Tumor diameter			
<40 mm	29(40.3)	43(59.7)	0.115
≥40 mm	52(52.5)	47(47.5)	
Depth of invasion			
Submucosa	6(50.0)	6(50.0)	0.782
Muscularis propria	28(48.3)	30(51.7)	
Adventitia	47(46.5)	54(53.5)	
pT classification			
T1 ~ T2	32(54.2)	27(45.8)	0.194
T3 ~ T4	49(43.8)	63(56.3)	
pN classification			
YES	30(39.0)	47(61.0)	0.047
NO	51(54.3)	43(45.7)	
pMetastasis			
YES	5(55.6)	4 (44.4)	0.616
NO	76(46.9)	86 (53.1)	

* The percentages in Table 1 are the row percentages.

### RNA extraction, reverse transcription (RT) and real-time PCR

Total RNAs from cells and primary tumor tissue were extracted using the Trizol reagent (Invitrogen) according to the manufacturer's instruction. The RNAs were pretreated with RNAase-free DNase and 2 μg RNA from each sample was used for cDNA synthesis with random hexamers. Real-time PCR was then employed to determine the fold change of *Bmi-1 *mRNA both in the primary esophageal tumor and in its paired normal esophageal tissue taken from the same patient. Expression data were normalized to the geometric mean by housekeeping gene Glyceraldehyde-3-phosphate dehydrogenase (*GAPDH*), which was used as an internal control. RT-PCR primers for *Bmi-1 *and *GAPDH *cDNA were as follows, *Bmi-1 *sense, 5'-ATGCATCGAACAACGAGAATCAAGATCACT-3'; *Bmi-1 *antisense, 5'-TCAACCAGAAGAAGTTGCTGATGACCC-3'; *GAPDH *sense, 5'-AATCCCATCACCATCTTCCA-3'; *GAPDH *antisense, 5'-CCTGCTTCACCACCTTCTTG-3'. The sequences of the primers and probe for real-time PCR of *Bmi-1 *and *GAPDH *were listed as follows, *Bmi-1 *sense: 5'-CTGGTTGCCCATTGACAGC-3'; *Bmi-1 *antisense: 5'-CAGAAAATGAATGCGAGCCA-3'; *GAPDH *sense: 5'-GACTCATGACCACAGTCCATGC-3'; *GAPDH *antisense: 5'-AGAGGCAGGGATGATGTTCTG-3'. The probes for *Bmi-1*, 5'- CAGCTCGCTTCAAGATGGCCGC-3', and for *GAPDH*, 5'-CATCACTGCCACCCAGAAGACTGTG-3', were labeled with 6-carboxy-fluorescein as the reporter dye.

### Western blot analysis

Western blot analysis of Bmi-1 expression was performed as described previously[16]. Bmi-1 was detected using a mouse monoclonal antibody against Bmi-1 (Upstate Biotechnology, Lake Placid, USA). An anti-α-tubulin mouse monoclonal antibody (1:1,000; Santa Cruz Biotechnology, Santa Cruz, CA) was used to confirm equal loading. Sera Bmi-1 autoantibodies were confirmed by Western blot analysis. Briefly, the recombinant Bmi-1 protein was separated by SDS-PAGE (1 μg/lane) and then blotted on PVDF. The membrane was cut into strips and incubated with 1 ml of serum samples diluted 1:100 in 3% skim dry milk solution in TBS. Strips were then incubated for 1 h with 1 ml of 1:2500 diluted goat F (ab')2 antihuman IgG labeled with horseradish peroxidase (Coulter Immunodiagnostics) in TBST and enhanced chemiluminescence.

### Immunohistochemistry

Immunohistochemistry was done to study altered protein expression in 171 human ESCC tissue,which was the same samples as our previous report[9]. The procedure was described previously[16]. Briefly, mouse monoclonal anti-Bmi-1 (1:150, Upstate Biotechnology, Lake Placid, USA) was incubated with the sections overnight at 4°C. For negative controls, the primary antibody was replaced by normal mouse serum. After washing, the tissue sections were treated with biotinylated anti-mouse secondary antibody (Zymed, San Francisco, CA), followed by further incubation with streptavidin HRP complex (Zymed). The degree of immunostaining of formalin-fixed, paraffin-embedded sections was reviewed and scored by two independent observers. The proportion of the stained cells and the extent of the staining were used as criteria of evaluation. For each case, at least 1,000 tumor cells were analyzed and the percentage of positively nuclear stained tumor cells was recorded. For each sample, the proportion of Bmi-1-expressing cells varied from 0% to 100%, and the intensity of nuclear staining varied from weak to strong. One score was given according to the percent of positive cells as: ≤5% of the cells: 1 point; 6-35% of the cells: 2 point; 36-70% of the cells: 3 point; ≥71% of the cells: 4 point. Another score was given according to the intensity of staining as negative staining: 1 point; weak staining (light yellow): 2 point; moderate staining (yellowish brown): 3 point; and strong staining (brown): 4 point. A final score was then calculated by multiple the above two scores. If the final score was equal or bigger than four, the tumor was considered high expression; otherwise, the tumor was considered low expression [9,27].

### Preparation of Recombinant Bmi-1 protein

GST-Bmi-1 construct was generated by subcloning the PCR-amplified human Bmi-1 coding sequence into pGEX-4T1. Overexpression in *Escherichia coli *and purification were performed according to the manufacturer's protocol (Amersham Pharmacia Biotech). The purified Bmi-1 protein was obtained by elution after cleavage. Purity of the recombinant protein was determined by SDS-PAGE and Coomassie Blue staining.

### ELISA

Purified recombinant Bmi-1 was diluted in 50 mM bicarbonate buffer (pH 9.5) to a final protein concentration of 5 mg/ml as determined by the Bradford assay (Bio-Rad Laboratories). The Bmi-1 solutions were dispensed into 96-well plates (100 μl/well) and incubated overnight at 4°C. The diluted serum samples (1:100 in PBST) were added at 100 μl per precoated well. Each well was determined with 100 μl of a 1:5,000 dilution of goat antihuman IgG-HRP conjugate (Santa Cruz). After a final PBST washing, TMB developing reagent was added for 15 min. Reaction was then stopped with 0.5 M H_2_SO_4 _and read at OD of 450 nm. All serum samples were run in duplicate and randomly distributed on the plates. Sera from cancer patients and sera from healthy volunteers were tested simultaneously.

### Statistical analysis

All statistical analyses were carried out using the SPSS 13.0 statistical software package. Mann-Whitney *U *test was used to analyze the relationship between Bmi-1 expression and clinicopathologic characteristics in archival esophageal cancer tissue and look for an association between Bmi-1 autoantibodies and clinicopathologic variables in blood samples from ESCC cancer patients. Pearson's chi-squared test was used to analyze the relationship between Bmi-1 expression and gender. Survival curves were plotted by the Kaplan-Meier method and compared by the log-rank test. The significance of various variables for survival was analyzed by the Cox proportional hazards model in the multivariate analysis. *P *< 0.05 in all cases was considered statistically significant.

## Results

### Expression of Bmi-1 in esophageal carcinoma cell lines

**Figure 1:**
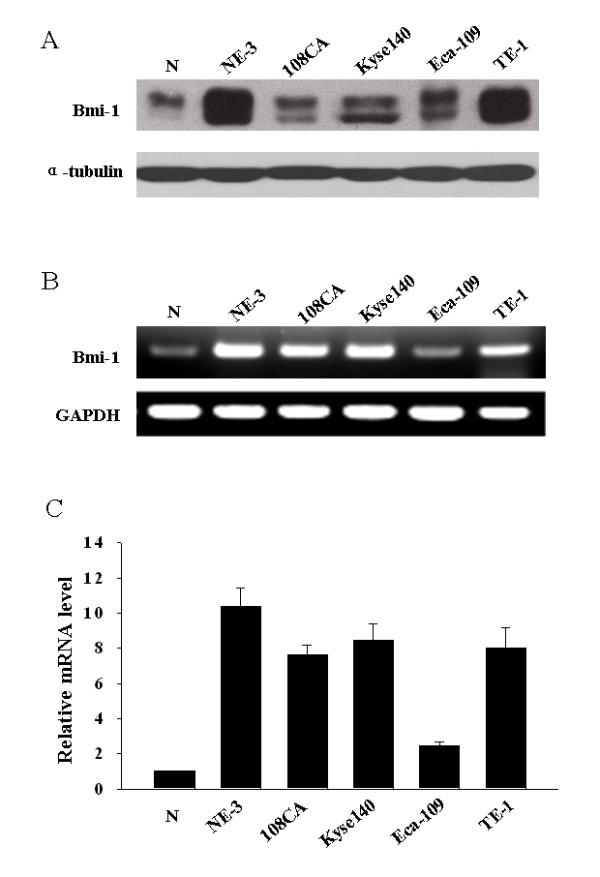
**Overexpression of Bmi-1 in immortalized esophageal epithelial cell line and esophageal carcinoma cell lines**. Expression of Bmi-1 protein **(A)**, mRNA (**B **and **C**) in immortalized esophageal epithelial cell line (NE-3) and esophageal carcinoma cell lines (108CA, Kyse 140, Eca-109 and TE-1) analyzed by Western blot (**A**), RT-PCR (**B**) or real-time PCR (**C**). N was a normal esophageal tissue. Expression level was normalized by α-Tubulin or GAPDH. Error bars represent standard deviations (SD) calculated from three parallel experiments.

Western blotting analysis showed that Bmi-1 protein was highly expressed in four esophageal cancer cell lines (108CA, Kyse 140, Eca-109 and TE-1) and HPV E6/E7 induced immortalized cell line NE-3, whereas it was weakly detected in normal esophageal tissue (Fig. 1A). The overexpression of Bmi-1 protein in ESCC cell lines (108CA, Kyse 140, Eca-109) was further confirmed when compared to that in the primary cultured esophageal epithelial cells from two independent donors (Additional file 1: Fig.S1). To determine whether the Bmi-1 upregulation was also at the mRNA level, RT-PCR and real-time PCR were performed. As shown in Fig. 1B and 1C, in parallel with the upregulated Bmi-1 protein, four cancer cell lines and the immortalized NE-3 cells unexceptionally showed high level expression of Bmi-1 mRNA, while the normal esophageal tissue expressed a relatively low level of Bmi-1 mRNA.

### Expression of Bmi-1 in paired esophageal tumor and nontumorous tissue

**Figure 2:**
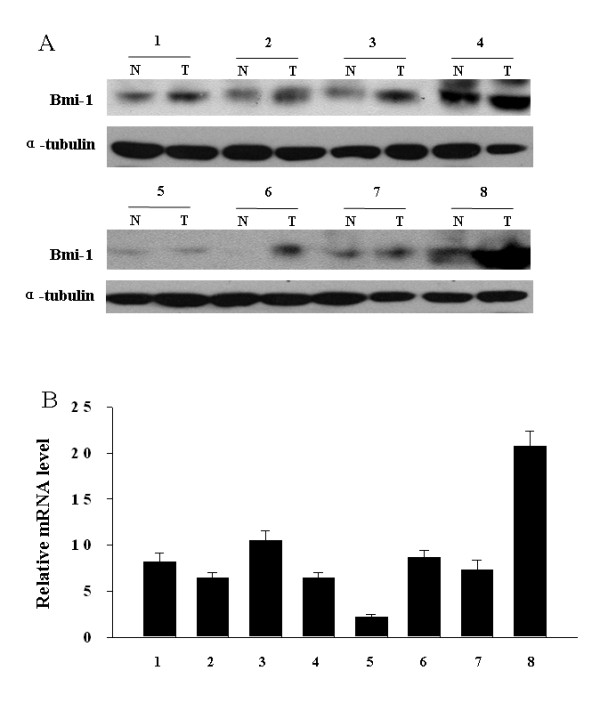
**Overexpression of Bmi-1 in ESCC tumors**. Eight paired primary esophageal tumors (T) and normal esophageal tissue (N) from the same patient were determined for Bmi-1 expression at protein level by Western blot **(A)**, or at mRNA level by real-time PCR **(B)**. Expression level was normalized by α-Tubulin or GAPDH. Error bars represent standard deviations (SD) calculated from three parallel experiments.

We next determined whether Bmi-1 is overexpressed in esophageal carcinoma samples. As shown in Fig. 2A, the expression level of Bmi-1 protein in cancer tissue was higher than that in the paired non-tumor tissues. Consistent with the upregulated protein level, Bmi-1 mRNA expression was also upregulated in tumor tissue compared with the paired non-tumor tissue as analyzed by real-time PCR (Fig. 2B). The tumor/normal (T/N) ratio of Bmi-1 message signals varied from approximately 2.5- to 20-fold in eight paired tissue. Thus, Bmi-1 is overexpressed at both mRNA and protein levels in the most of eight pairs of ESCC tumors examined.

### Expression of Bmi-1 in archival esophageal cancer tissue

**Figure 3:**
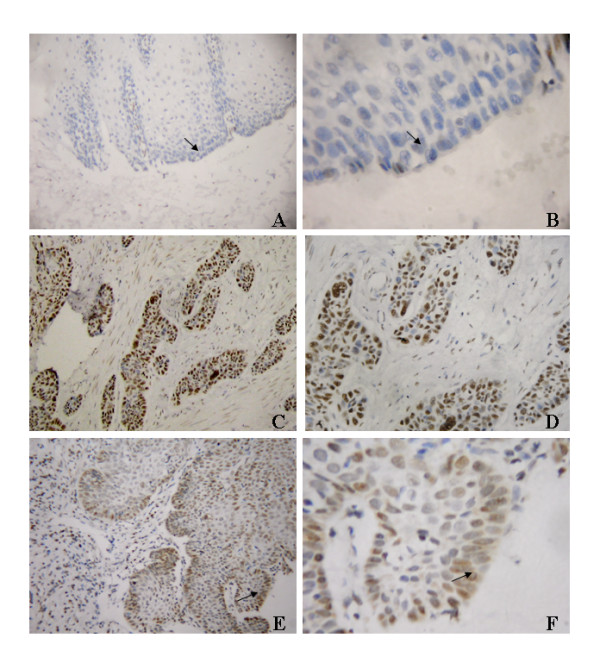
**Expression of Bmi-1 protein in tissue by immunohistochemistry**. **A **and **B**, only weak staining of Bmi-1 was detected in few normal esophageal epithelial tissue (arrow, normal epithelial cells). **C **and **D**, positive expression of Bmi-1 in esophageal carcinoma tissues (200× and 400× of magnification, respectively). **E **and **F**, representative case of higher expression of Bmi-1 in invasive front (arrow, 200× and 400× magnification, respectively). Bmi-1 expression mainly localized in nuclei of tumor cells.

We further analyzed the expression and subcellular localization of Bmi-1 protein by immunohistochemistry. Bmi-1 protein was detected in 110 of 171 ESCC samples (64.3%). Bmi-1 protein was mainly located in nuclei of tumor cells (Fig.3C and 3D). Higher expression of Bmi-1 was observed in invasive front of esophageal carcinoma tissue (Fig.3E and 3F). However, only weak Bmi-1 staining was observed in some basal cells of adjacent normal esophageal epithelium (Fig. 3A and 3B) and in few surrounding stroma cells.

However, the results of RT-PCR and Western blot in the 8 paired fresh tissues were not consistent with the results of immunostaining in the archive tissue samples. To clarify the discrepancy, immunohistochemical analysis of the 8 paired tissues was carried out. The representative results were shown in Additional File 2: Fig.S2. Case 4 and case 8 contained high amounts of tumor cells with high levels of Bmi-1 expression. Case 1, case 2 and case 3 showed relatively low amounts of tumor cells with high levels of Bmi-1 expression, whereas case 5, case 6 and case 7 showed low level of Bmi-1 expression in tumor cells

### Correlation between Bmi-1 protein expression and clinicopathological features

Table 1 shows the relationship between the expression of Bmi-1 protein and clinical characteristics in 171 ESCC cases. There was no significant correlation between the expression level of Bmi-1 protein and age, histological classification, histological differentiation, tumor diameter, depth of invasion, T classification or distant metastasis of esophageal cancer patients. Due to the number of cases with stage I, IIb and IV is small and a relatively high frequent expression in patients with IIb in comparison to IIa and I, we combined patients into two groups according the stage, one group with stage I and IIa, and the other group with stage IIb, III and IV. There is significant difference of Bmi-1expression between the early stages (I plus IIa) and other stages (IIb-IV) (*P *= 0.003). In addition, the expression of Bmi-1 is closely associated with pN classification (*P *= 0.047). The expression of Bmi-1 protein was positively correlated with staging and pN classification (Table 1). Higher staging and N classification correlated with higher Bmi-1 expression.

### Survival analysis

**Figure 4:**
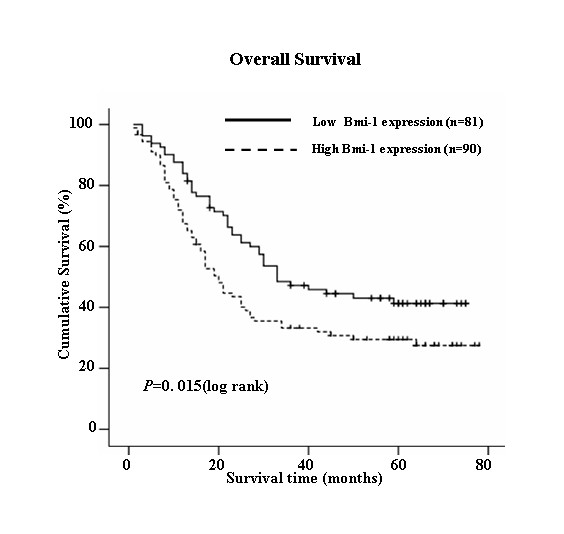
**Kaplan-Meier curves with univariate analyses (log-rank) for patients with low Bmi-1 expression versus high Bmi-1 expression tumors**. The cumulative 5-y survival rate was 41.3% in the low Bmi-1 protein expression group (n = 81) (bold line), but it was only 33.2% in the high expression group (n = 91) (dotted line) (P < 0.05, Log-rank).

Kaplan-Meier analysis and the log-rank test were used to evaluate the effect of classic clinicopathological characteristics (including gender, stage, N classification) and Bmi-1 expression on survival. The expression level of Bmi-1 protein in esophageal carcinoma was significantly correlated with patients' survival time (*P *= 0.015), that was the higher level of Bmi-1 expression correlated with shorter survival time. As shown in Fig. 4, the cumulative 5-year survival rate was 41.3% in the low Bmi-1 expression group, whereas it was only 33.2% in the high Bmi-1 expression group (*P *= 0.015, Log-rank).

In addition, N classification, stage and gender were also significantly correlated with survival in Kaplan-Meier analysis and log-rank test (for N classification, *P *< 0.001; for stage, *P *= 0.039 and for gender, *P *= 0.005). But age was not correlated with survival in Kaplan-Meier analysis and log-rank test (*P *> 0.05). We did multivariate survival analysis, which included Bmi-1 expression level, age, stage, N classification and gender, to determine if Bmi-1 expression level was an independent prognostic factor of outcomes. In this analysis, N classification, gender and Bmi-1 expression were recognized as independent prognostic factors (Table 2). Thus, our findings indicate that Bmi-1 protein expression level has a significant correlation with prognosis of esophageal carcinoma.

**Table 2:** Univariate and multivariate analysis of different prognostic parameters in patients with esophageal carcinoma by Cox-regression analysis

	Univariate analysis		Multivariate analysis			
	
	No. patients	p	Regression coefficient(SE)	p	Relative risk	95% confidence interval
Age						
<60	103	0.071	0.201(0.196)	0.306	1.222	0.832~1.794
≥60	68					
pN metastasis			0.631(0.195)	0.001	1.879	1.283~2.752
Yes	77	<0.001				
No	94					
Stage			-0.347(0.294)	0.237	0.707	0.398~1.257
I-II	99	0.039				
III-IV	72					
Gender			-0.631(0.253)	0.013	0.532	0.324~0.874
Male	129	0.005				
Female	42					
Bmi-1 expression			0.401(0.195)	0.040	1.501	1.022~2.205
No or low	81	0.015				
High	90					

**Figure 5:**
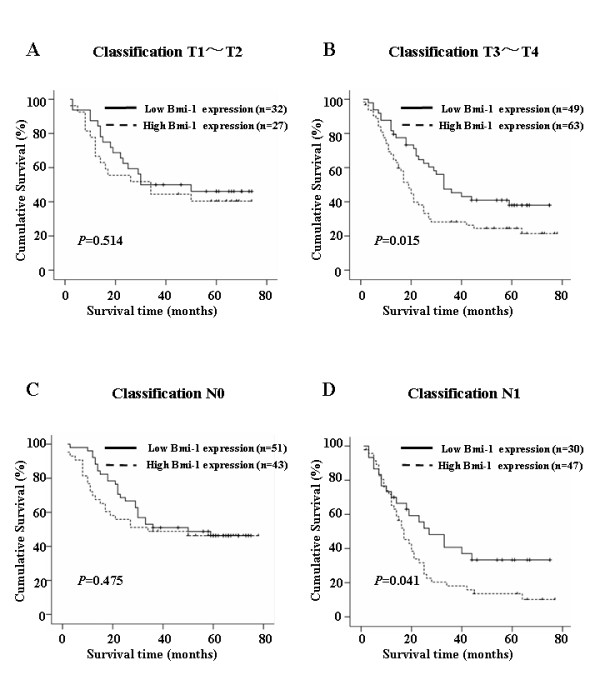
**Kaplan-Meier analysis of the overall survival of esophageal carcinoma patients categorized according to the T or N classification and status of Bmi-1 expression**. The statistical difference of Bmi-1 high-expressing and low-expressing patients was compared between T1-T2 **(A) **and T3-T4 **(B) **subgroups. The same comparison was carried out in N0 **(C) **and N1 **(D) groups**. P values were calculated by the log-rank test.

We also analyzed the prognostic value of Bmi-1 expression in selective patient subgroups stratified according to the stage, T and N classification, respectively. Patients with tumors exhibiting high Bmi-1 expression had significantly shorter overall survival compared with patients with low expression of Bmi-1 in the T3-T4 subgroup (n = 112; log-rank, *P *= 0.015; Fig. 5B) and the N1 subgroup (n = 77; log-rank, *P *= 0.041; Fig. 5D). A similar analysis of the T1-T2 subgroups (n = 59; log-rank, *P *= 0.514; Fig. 5A) and the N0 subgroup (n = 94; log-rank, *P *= 0.475; Fig. 5C) did not show statistically significant differences between patients with different Bmi-1 expression level.

### Detection of Anti-Bmi-1 Antibodies by ELISA

**Figure 6:**
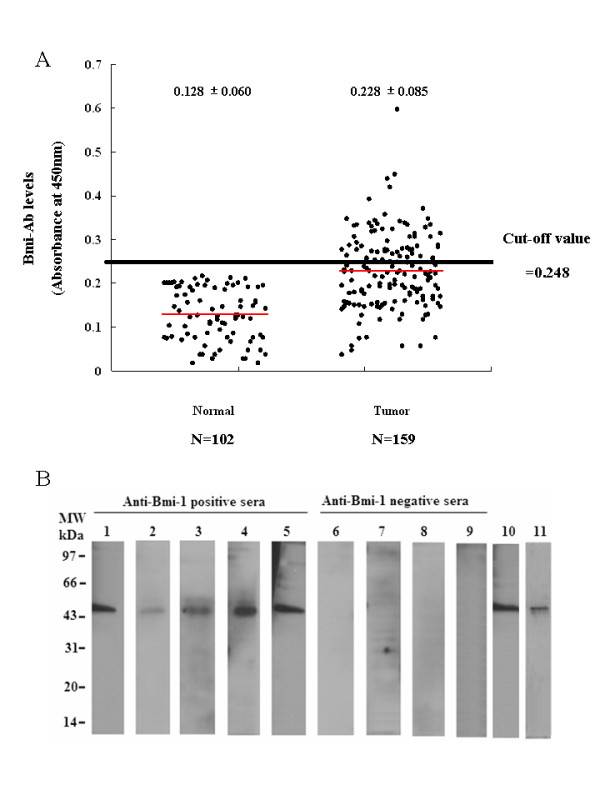
**Detection of Bmi-1 antibody in sera from esophageal cancer patients**. **(A) **Absorbance ratios in anti-Bmi-1 ELISA for sera from healthy control (Normal) and patients with ESCC (Tumor). The cutoff for positive antibody reactivity against Bmi-1 was 0.248, which was defined as an absorbance greater than 2 SDs above the mean value of the control. **(B) **Reactivity of sera from cancer patients with recombinant Bmi-1 protein in Western blotting. Lanes 1-9, stained with 1:100 diluted sera (1 μg Bmi-1/lane). Lanes 1-5, sera from esophageal cancer patients; Lanes 6-9, sera from healthy volunteers; Lane 10. staining of Bmi-1 by anti-Bmi-1 monoclonal antibody (1 μg Bmi-1/lane); Lane 11, staining of purified recombinant Bmi-1 protein (5 μg Bmi-1/lane)by Coomassie blue staining.

We asked whether overexpression of Bmi-1 could activate immune system to produce Bmi-1 autoantibodies. Bmi-1 autoantibodies were determined by ELISA using purified recombinant Bmi-1 antigen. The mean (SD) absorbance ratio was 0.128 (0.060) in sera from control (n = 102) and 0.228 (0.085) in sera from esophageal cancer patients (n = 159) (Fig. 6A). Bmi-1 autoantibodies in sera from ESCC patients was significantly greater than in healthy controls (*P *< 0.001). The cutoff for positive antibody reactivity against Bmi-1 was 0.248, which was defined as an absorbance greater than 2 SDs above the mean value of the control. Sera from 62 of 159 esophageal cancer patients (39.0%) were reactive with recombinant Bmi-1 in ELISA, whereas none of the control sera from healthy volunteers recognized Bmi-1 (Table 3). The relationship between anti-Bmi-1 autoantibody and clinicopathological variables is shown in Table 3. Anti-Bmi-1 antibodies were not statistically associated with T classification or metastasis. However, correlations were significant between anti-Bmi-1 and tumor stage (*P *= 0.040), and lymph node status (N classification; *P *< 0.001). There was a higher incidence of Bmi-1 antibodies in the advanced disease group than in the early disease group. Bmi-1 antibodies were also significantly much prevalent in patients with lymph node metastasis than those in patients without lymph node metastasis.

**Table 3:** Relationships of the presence of anti-Bmi-1 autoantibody with clinicopathologic variables.

			Anti-Bmi-1		
				
Characteristics	Total (n = 159)	OD ± SD	Negative cases 61.0%	Positive cases 39.0%	P
Stage					0.040
I	6	0.1883 ± 0.0459	6(100)	0(0.0)	
II	72	0.2274 ± 0.0795	46(63.9)	26(36.1)	
III	69	0.2394 ± 0.0932	39(56.5)	30(43.5)	
IV	12	0.2441 ± 0.0755	5(41.7)	7(58.3)	
pT classification					0.747
T1	9	0.1983 ± 0.0552	8(88.9)	1(11.1)	
T2	40	0.2312 ± 0.0815	22(55.0)	18(45.0)	
T3	80	0.2389 ± 0.0857	49(61.2)	31(38.8)	
T4	30	0.2230 ± 0.0927	18(60.0)	12(40.0)	
pN classification					<0.001
YES	75	0.2596 ± 0.0826	35(46.7)	40(53.3)	
NO	84	0.2067 ± 0.0787	62(73.8)	22(26.2)	
pMetastasis					0.170
YES	12	0.2441 ± 0.0755	5(41.7)	7(58.3)	
NO	147	0.2385 ± 0.0858	91(61.9)	56(38.1)	

### Detection of Bmi-1 autoantibodies in sera from patients with ESCC by Immunoblotting

The 62 sera recognizing Bmi-1 in the ELISA were tested against recombinant denatured Bmi-1 protein by immunoblotting. Under the conditions used, all of 62 sera (100%) recognized the Mr 41,000 denatured recombinant protein Bmi-1 in Western blot analysis. No reactivity was found in any of 20 control sera selected from healthy volunteers. To illustrate the immunoblot analysis, Fig. 6B shows the staining pattern of representatively positive sera from esophageal cancer patients and healthy control. In addition, staining of blotted Bmi-1 by an anti-Bmi-1 monoclonal antibody and purified recombinant Bmi-1 protein were also included.

## Discussion

Here, we presented Bmi-1 was upregulated in ESCC and the expression of Bmi-1 in ESCC was mainly in nuclei of tumor cells, which was in accordance with the findings in the studies of other cancers [16,24]. We reported the elevated Bmi-1 expression was correlated with the stage and pN classification of the disease and poor prognosis of patients. In addition, the presence of Bmi-1 autoantibodies in sera from patients with ESCC may have clinical utility in esophageal cancer screening, diagnosis and prediction of lymph node metastasis. Taken together, our study suggests that Bmi-1 might represent a novel indicator for the prognosis of ESCC patients.

Consistent with our previous studies in other types of cancer[16,24], we found that Bmi-1 was overexpressed in immortalized esophageal epithelial cells and ESCC cell lines as well as in ESCC tissue both at transcriptional and translational level. The accumulation of multiple genetic alterations may be required over a long period of time during the development and progression of ESCC[28]. Cell immortalization is the ability of normal cells to grow through an indefinite number of divisions in culture[29]. Immortalized cells are capable of unlimited proliferation and represent the early stage of transformation before full malignant transformation. Our results suggest that Bmi-1 may be an early transformation factor of esophageal epithelial cells. It has been reported that overexpression of Bmi-1 alone was able to immortalized human mammary epithelial cells (HMEC) and nasopharyngeal epithelial cells (NPEC) [16,24]. Thus, it will be important to determine whether overexpression of Bmi-1 lead to immortalization of esophageal epithelial cells.

ESCC shows a poor prognosis because of the occurrence of systemic metastasis, mainly via lymphatic vessels[30-32]. Various proteins have been shown to be associated with development and progression of ESCC, including cyclin D1[5], Ki-67[6], nm23-H1[7] and Fas[8]. We have shown that the expression of Bmi-1 is closely associated with advanced stage and lymph node metastatic status of esophageal cancer patients, which is strongly suggesting that Bmi-1 can be used as a marker to identify subsets of ESCC cancer patients with more aggressive feature. It suggests that Bmi-1 protein may play a role in tumor metastasis, especially in lymph node metastasis. As determined in the same set of samples, our previously study showed that CENP-H was overexpressed in ESCC[9]. However, there was no significant correlation between the expression level of Bmi-1 protein and CENP-H protein (*P *= 0.085), though a close examination of co-expression of the two proteins on survival would be further analyzed in the future. Importantly, patients with higher Bmi-1 expression had shorter overall survival time, whereas patients with lower Bmi-1 expression had better survival, and Bmi-1 expression was identified as an independent prognostic factor. These results are consistent with a recent report about Bmi-1 mRNA expression in ESCC by He, et al [25]. However, the authors failed to find a clinical relevance with the expression of Bmi-1 protein. The discrepancy between our finding and He's finding may due to the different antibodies used, or different clinical subjects used in the two studies. Consistent with other reports, the anti-Bmi-1 antibody used in our study was tumor cell nucleus staining, but contrast to He's report in which Bmi-1 mainly observed in tumor cell cytoplasm less or not in nucleus. In addition, we found that in subgroup of patient with T classification as T3-T4 or N classification as N1, higher Bmi-1 expression also indicated a shorter overall survival time. These results indicate Bmi-1 is a predictor of lymph node metastasis and may play a more important role in late stage ESCC. However, a study in an independent cohort of samples is required to confirm our findings.

Consistent with a recent report, which showed that Bmi-1 autoantibodies in sera were a potential new biomarker of nasopharyngeal carcinoma [17], our results showed that Bmi-1 autoantibodies were presented in a subgroup of ESCC sera. Anti-Bmi-1 was significantly correlated with tumor stage (P = 0.040), and lymph node status (N classification; P < 0.001). There was a strong correlation between Bmi-1 immunostaining and the presence of serum Bmi-1-Abs (Additional file 3: Table S1). It indicates that Bmi-1 antibodies are more prevalent in sera from patients with later stage tumor than in sera from patients with early stage tumor. This may be attributed to higher expression of Bmi-1 in cancer cells from later stage tumor tissue than from early stage tumor tissue. There was also higher incidence of Bmi-1 antibodies in sera from patients with lymph node metastasis than without lymph node metastasis. It is possible that the power of massive lymphocytes closely contacting with invasive tumor cells, may augment the immunoreactive to Bmi-1 antigen, which facilitates the production of Bmi-1 antibodies. Thus, Bmi-1 antibodies are a novel potential biomarker for ESCC, but it requires further investigation to determine whether Bmi-1 antibodies could be used as prognostic marker. In ESCC, autoimmunity has been shown against several proteins, including cytokeratins[33], p53[34], TRIM21[35], myomegalin[36], peroxiredoxin VI protein[37] and CDC25b[38]. However, systemic investigations are required before clinical utility of the autoantibodies in diagnosis or prognosis.

## Conclusion

This is the first study showing the expression of Bmi-1 in esophageal cancer cell lines as well as tumor tissue, highlighting the clinical significance of Bmi-1 in esophageal carcinoma. The presence of Bmi-1 autoantibodies in sera from patients with ESCC may have potential clinical utility in esophageal cancer diagnosis. Bmi-1 might be used as a valuable prognostic marker for esophageal carcinoma patients. However, further studies are needed to clarify the mechanism by which Bmi-1 is involved in the development and progression of esophageal carcinoma and its exact role in the regulation of carcinogenesis in esophageal carcinoma.

## Competing interests

The authors declare that they have no competing interests.

## Authors' contributions

XZG and WLL were responsible for data collection and analysis, experiment job, interpretation of the results, and writing the manuscript. LJZ, GZ, LBS, LHX were responsible for conducting the data analysis in cooperation with JYW, SG and YMC. QLK, MZL were responsible for reviewing and scoring the degree of immunostaining of sections. MSZ was responsible for experimental design, analysis and interpretation. All authors have read and approved the final manuscript.

## Pre-publication history

The pre-publication history for this paper can be accessed here:

http://www.biomedcentral.com/1471-2407/10/467/prepub

## Supplementary Material

Additional file 1 **Expression of Bmi-1 in primary normal esophageal epithelial cells (NEECs) and esophageal carcinoma cell lines by Western blot analysis**. The expression of Bmi-1 protein in ESCC cell lines (108CA, Kyse 140, Eca-109) and the primary cultured normal esophageal epithelial cells from two independent donors analyzed by Western blot was shown in this figure. **A**. Primary culture of NEEC (the arrow shows a piece of biopsy). **B**. Western blot analysis of E-cadherin in NEEC1, NEEC2. α-Tubulin was used as a loading control. **C**. Western blot analysis of Bmi-1 in NEEC1, NEEC2 and various esophageal carcinoma cell lines. α-Tubulin was used as a loading control. Click here for file

Additional file 2 **Expression of Bmi-1 in the 8 paired fresh tissue samples by immunohistochemical analysis**. Representative immunohistochemistry staining for Bmi-1 protein in the 8 pairs of ESCC specimens used in western blot assay was shown in this figure (Original magnification, 200×). Click here for file

Additional file 3 **The correlation between Bmi-1 immunostaining and the presence of serum Bmi-1-Abs**. The correlation between Bmi-1 immunostaining in 40 tumor samples and the presence of serum Bmi-1-Abs in those matched serum samples was shown in this table. Click here for file
