# Supplementary figures and images for: Prognostic relevance of Bmi-1 expression and autoantibodies in esophageal squamous cell carcinoma
Source: BMC Cancer. 2010 Sep 1;10:467. doi: 10.1186/1471-2407-10-467 (PMC2942852; doi:10.1186/1471-2407-10-467)

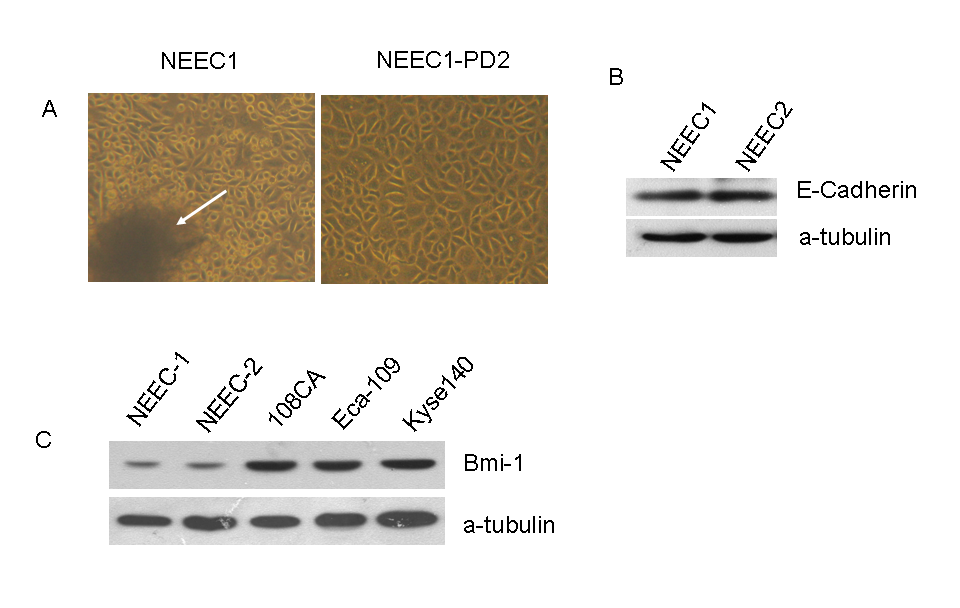

Supplement: Additional file 1 — Expression of Bmi-1 in primary normal esophageal epithelial cells (NEECs) and esophageal carcinoma cell lines by Western blot analysis. The expression of Bmi-1 protein in ESCC cell lines (108CA, Kyse 140, Eca-109) and the primary cultured normal esophageal epithelial cells from two independent donors analyzed by Western blot was shown in this figure. A. Primary culture of NEEC (the arrow shows a piece of biopsy). B. Western blot analysis of E-cadherin in NEEC1, NEEC2. α-Tubulin was used as a loading control. C. Western blot analysis of Bmi-1 in NEEC1, NEEC2 and various esophageal carcinoma cell lines. α-Tubulin was used as a loading control. [file 1471-2407-10-467-S1.TIFF]

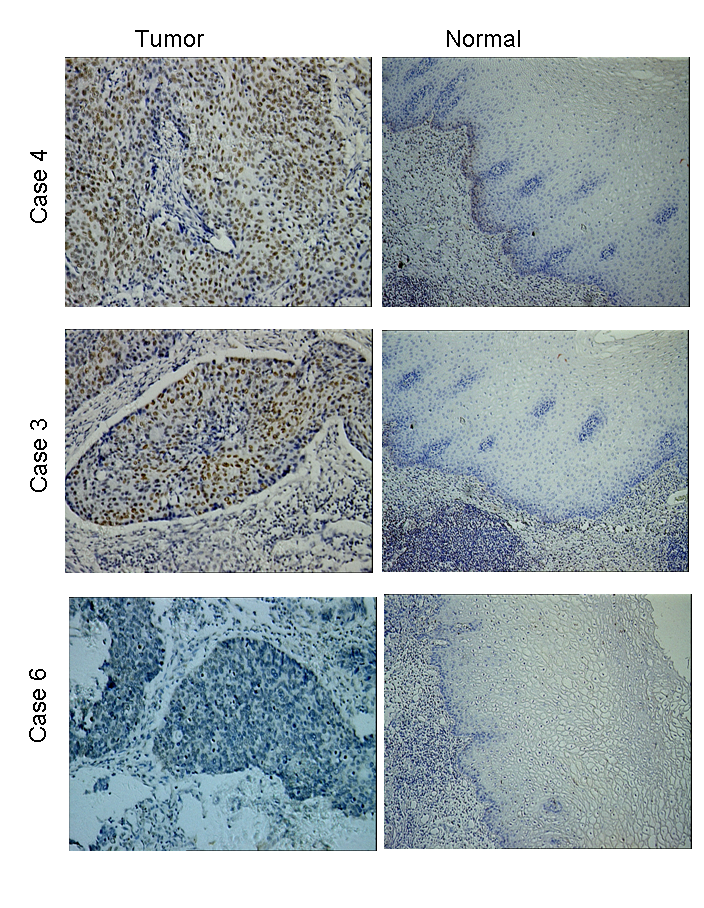

Supplement: Additional file 2 — Expression of Bmi-1 in the 8 paired fresh tissue samples by immunohistochemical analysis. Representative immunohistochemistry staining for Bmi-1 protein in the 8 pairs of ESCC specimens used in western blot assay was shown in this figure (Original magnification, 200×). [file 1471-2407-10-467-S2.TIFF]
