# Supplementary material for: Prognostic relevance of Bmi-1 expression and autoantibodies in esophageal squamous cell carcinoma
Source: BMC Cancer. 2010 Sep 1;10:467. doi: 10.1186/1471-2407-10-467 (PMC2942852; doi:10.1186/1471-2407-10-467)
Supplement: Additional file 3 — The correlation between Bmi-1 immunostaining and the presence of serum Bmi-1-Abs. The correlation between Bmi-1 immunostaining in 40 tumor samples and the presence of serum Bmi-1-Abs in those matched serum samples was shown in this table. [file 1471-2407-10-467-S3.DOC]

**The correlation between Bmi-1 immunostaining and the presence of serum Bmi-1-Abs.**

**Methods**

**Serum samples and formalin-fixed samples.** To investigate the correlation between Bmi-1 staining and autoantibodies, serum samples were obtained with informed consent from 94 ESCC patients admitted to the cancer center of Sun Yet-sen university from January 2009 to December 2009 (27 females and 67 males; median age 64.0 with a range of 49 - 81). Samples were selected for the study on the basis of the following criteria: (1) patients were newly diagnosed and previously untreated and (2) their tumors were pathologically diagnosed as ESCC (stages II - IV). Serum was obtained at the time of diagnosis and stored at -80°C. After analysis of autoantibodies, formalin-fixed tumor specimens were obtained from 40 patients including 20 Bmi-1 autoantibodies positive patients and 20 Bmi-1 autoantibodies negative patients (11 female and 29 male patients; median age of 63.0 with a range of 61 - 72 years) were selected from the above mentioned 94 patients for Bmi-1 immunostaining. These ESCC patients received resection of their primary cancers without preoperative chemo- and/or radiotherapy. The pathological stage was determined according to the pathological TNM classification. Prior to the use of these clinical materials for investigation, informed consent from patients and approval from the Institute Research Ethics Committee were obtained.

**Statistical analysis.** Mann-Whitney U test was used to analyze the relationship between Bmi-1 staining and the presence of serum Bmi-1 autoantibodies.

**Results**

**Relation between the presence of Bmi-1 autoantibodies and Bmi-1 immunostaining.** Bmi-1 autoantibodies were analyzed by ELISA. The mean (SD) absorbance ratio was 0.219 (0.071) in sera from esophageal cancer patients (n=94). According to the cutoff value of 0.248, sera from 38 of 94 esophageal cancer patients (36.7%) were reactive with recombinant Bmi-1 in ELISA.

Matched formalin-fixed tumor specimens from 20 Bmi-1 autoantibodies positive patients and 20 Bmi-1 autoantibodies negative patients were used to determine the relation between the presence of Bmi-1 autoantibodies and Bmi-1 immunostaining. The expression of Bmi-1 protein was analyzed by immuohistochemistry. As shown in supplemental Table S1, high Bmi-1 expression was found in 19 of 20 (95%) seropositive patients and 2 of 20 (10%) seronegative patients, no or low Bmi-1 expression was found in 1 of 20 (5%) seropositive patients and 18 of 20 (90%) seronegative patients. There was a strong correlation between Bmi-1 immunostaining and the presence of Bmi-1 autoantibodies (*P*<0.001).

**Table S1. Correlation between the presence of Bmi-1 autoantibodies and Bmi-1 immunosataining**

| **Bmi-1**  **Immunostaining** | **Serum Bmi-1 autoantibodies** |
| --- | --- |
|  | **Positive Negative**  **(n=) (n=) *P*** |
| High expression (%) 19 (95%) 2 (10%) _  Low and no expression (%) 1 (5%) 18 (90%) <0.001 | |
